# Diabetic Ketoacidosis Post Bariatric Surgery

**DOI:** 10.3389/fendo.2018.00812

**Published:** 2019-01-15

**Authors:** Ivania M. Rizo, Caroline M. Apovian

**Affiliations:** Section of Endocrinology, Diabetes, Nutrition, and Weight Management, Boston University School of Medicine and Boston Medical Center, Boston, MA, United States

**Keywords:** bariatric surgery, diabetic ketoacidosis, type 2 diabetes, Roux-en-Y gastric bypass, ketosis-prone diabetes mellitus

## Abstract

In patients with type 2 diabetes, bariatric surgery can lead to significant improvements in glycemic control and diabetes remission. We present a case of a Hispanic female with type 2 diabetes phenotype who underwent bariatric surgery and post-operatively stopped her insulin therapy due to multiple reasons, including decreased oral intake and concern for hypoglycemia. Ultimately, she developed diabetic ketoacidosis. She does not fit into the classical type 2 diabetes or type 1 diabetes definition but into the heterogeneous subgroup of diabetes called ketosis-prone diabetes.

## Introduction

Diabetes mellitus is a heterogeneous group of metabolic conditions all linked together by hyperglycemia. It primarily falls into two broad categories: type 1 and type 2. The most prevalent category, type 2 diabetes (T2D), is defined by a combination of resistance to insulin action and an inadequate compensatory insulin secretory response (1). Type 1 diabetes (T1D) results in an absolute deficiency of insulin secretion. It can be identified with certain background genetic susceptibility and serological evidence of an autoimmune T-cell mediated pathological process occurring in the pancreatic islets. Diabetic ketoacidosis (DKA) has been considered a key clinical feature of T1D although it has been reported in subjects who lack the T1D phenotype. An additional subgroup of diabetes has been emerging called ketosis-prone diabetes (KPDM). KPDM is a heterogeneous syndrome characterized by patients who present with DKA or unprovoked ketosis but do not necessarily have the phenotype of autoimmune type 1 diabetes. Most patients with KPDM are overweight or obese and usually present with a short history of hyperglycemic symptoms (2).

DKA is one of the most serious complications associated with diabetes. It is defined as a triad of uncontrolled hyperglycemia, metabolic acidosis, and increased total body ketones (3). Serum glucose is invariably elevated, barring pregnancy, or concurrent use of sodium-glucose co-transporter 2 (SGLT2) inhibitor, where glucose may be relatively normal. The metabolic abnormalities seen in DKA result from a relative deficiency in insulin combined with an increase in counter-regulatory hormones such as glucagon, catecholamines, growth hormone, and cortisol.

Bariatric surgery has emerged as a valuable tool for the treatment of diabetes and obesity. Bariatric surgery results in significant weight loss and may result in T2D remission. Mingrone et al. demonstrated a 75% remission in T2D 2 years status post RYGB or biliopancreatic diversion (4). Although it is also used in T1D and obesity with significant reduction in insulin requirements and modest improvements in hemoglobin A1c, those with T1D would not be able to stop insulin post-operatively. Prior to bariatric surgery it is essential to identify individuals with diabetes who will not be able to stop insulin despite significant weight loss or decreased oral intake.

## Case Presentation

Thirty-year-old Hispanic female with past medical history of T2D, hypertension, and Roux-en-Y (RYGB) gastric bypass presented to an academic urban medical center emergency room with nausea and abdominal pain and was found to have DKA.

The patient was diagnosed with T2D at age 15, while being hospitalized for an unrelated matter. Notably, at the time of diagnosis, she had obesity with a BMI of 56.4 kilograms per square meter (kg/m^2^). Insulin therapy was initiated at this time. The patient retrospectively reported being non-compliant with the insulin regimen since diagnosis and never developed DKA prior to this hospital admission. The struggle with diabetes was common in her family as her mother and two sisters are reported to have T2D.

The patient underwent laparoscopic RYGB gastric bypass 4 months prior to DKA presentation. Prior to RYGB, the patient was on Glargine 30 units per day, Lispro 15 units before meals, metformin 1,000 mg twice daily and empagliflozin 10 mg per day. Hemoglobin A1c was 9.9% 1 month prior to bariatric surgery. Patient was placed on an insulin drip intraoperatively and on post-operative day 0, insulin drip which was turned off when point of care glucose was 188 mg/dL. She was transitioned to Glargine 16 units per day and low dose insulin sliding scale that consisted of 1 unit of Lispro with meals for every 50 mg/dL glucose above 150 mg/dL. Nutritional insulin was held because the patient was on a clear liquid diet. On post-operative day 2, she was transitioned to high protein full liquid meals. The patient was discharged from the hospital on Glargine 10 units per day and metformin 1,000 mg twice a day. Empagliflozin was discontinued post operatively.

Two weeks after laparoscopic gastric bypass, the patient was seen in the bariatric clinic. She was tolerating the high protein full liquid diet without difficulty. She was taking Glargine 10 units once a day as prescribed and fasting blood glucose values were ranging from 80 to 125 mg/dL. At the time of her clinic visit, her diet was advanced to soft and moist proteins. The patient did not follow up at her scheduled 6-week post-operative appointment. Two months later, she presented to the emergency room with DKA.

At the emergency room, she reported 8 days of fevers, nausea, vomiting, poor oral intake, cough, and dizziness. She had stopped taking Glargine ~2 months prior to DKA presentation due to decreased oral intake and fear of hypoglycemia, although no hypoglycemia was recorded. She noted polyuria and polydipsia for some time. However, as her glucometer had broken, she had not obtained any blood glucose values. Otherwise, she had felt well until 8 days prior to presentation to emergency room. During that time, she reported a fever up to 105°F, and there were sick contacts in her house including small children with upper respiratory infection. The patient also noted difficulty tolerating solid foods. As a result, she was primarily eating protein shakes and liquids.

Initial labs were significant for hyperglycemia with blood glucose of 504 mg/dL, creatinine of 1.4 mg/dL, bicarbonate of 6 mmol/L, anion gap of 23 mEq/L, pH of 7.09, and urine ketones 2+ (Table 1). Her hemoglobin A1c was 9.2% at time of admission. Her Urinalysis and urine culture were not positive, comprehensive respiratory panel was not significant and Chest Xray at time of admission did not find any significant findings. At time of admission she had lost 17.27 kg since bariatric surgery, placing her at 78 kg and BMI of 34.84 kg/m^2^. She was placed on continuous insulin infusion and fluid hydration per the DKA insulin drip protocol. After 24 hours, she was transitioned from the insulin drip to a basal- bolus insulin regimen. Ultimately, she was discharged on Glargine 20 units once a day and Lispro 6 units with meals. All KPDM patients should be discharged from initial DKA admission on insulin for safety reasons. The need for insulin can be assessed in the weeks post-discharge.

**Table 1:** Initial laboratory values.

	**Laboratory**	**Patient Values (Normal Ranges)**
Venous blood gas	pH	7.09 (7.32–7.42)
	PCO2	24 mm Hg (35–45 mmHg)
	PO2	40 mmHg (30–50 mmHg)
	Bicarbonate	7.0 mmol/L (22–29 mmHg)
	Lactate	1.8 mmol/L (0.9–1.7 mmol/L)
Chemistry	Blood glucose	504 mg/dL (70–100 mg/dL)
	Sodium	128 mmol/L (135–145 mmol/L)
	Potassium	4.8 mmol/L (3.1–5.3 mmol/L)
	Chloride	99 mmol/L (98–110 mmol/L)
	Creatinine	1.4 mg/dL (0.5–1.1mg/dL)
	Glomerular filtration rate	44 mL/ min/1.73 m2 (>60 mL/ min/1.73m2)
	Albumin	4.6 g/dL (3.5–5.0 g/dL)
Hematology	Hemoglobin	15.7 g/dL (11.8–16.0g/dL)
	White blood cells	26.5 K/UL (4.0–11.0 K/UL)
	Platelets	352 K/UL (150–400 K/UL)

Sixteen days after discharge from the hospital, the patient was seen in the outpatient Endocrine clinic. She was doing well other than reporting a dry cough. We performed laboratory analysis which revealed concurrent blood glucose of 196 mg/dL and C-peptide of 1.36 ng/mL, glutamic acid de- carboxylase-65 antibody <5 IU/mL (anti-GAD), islet cell antibody negative, and Hemoglobin A1c of 9.3%. Zinc transporter 8 antibody was not checked.

## Discussion

In this case report, we describe a Hispanic female with phenotypic early onset T2D associated with class 3 obesity who had undergone RYGB gastric bypass and presented 3 months post-bariatric surgery with DKA. Our patient had overlapping features between types 1 and 2 diabetes. She had phenotypic T2D obesity, high risk ethnicity for T2D, and abdominal adiposity but developed ketoacidosis. Her subtype of diabetes does not clearly fit a type 1 or 2 diabetes definition and is most consistent with ketosis-prone diabetes mellitus (KPDM).

It was initially thought that most cases of KPDM present in African persons and in African-American individuals in the United States, but KPDM has been reported in Hispanics such as our patient (5). Several case series have reported that up to half of African-Americans and Hispanics hospitalized with DKA have a clinical presentation compatible with KPDM (6). Most patients with KPDM are overweight or obese with newly diagnosed diabetes or an acute course of hyperglycemia. As was seen in our patient, there is often a strong family history of T2D.

KPDM is a heterogeneous form of diabetes. It can be classified by the presence or absence of autoantibodies and the presence or absence of β- cell functional reserve (Aβ classification) (7). Individuals with islet cell autoantibodies are classified as A+ and those without A–. In addition, individuals with β- cell reserve, once the period of acute metabolic decompensation has resolved are labeled β+ vs. β- if there is no beta cell reserve. The largest subgroup of KPDM is the A–β+ (negative autoantibodies and positive beta cell reserve). Our patient had negative antibodies with β cell reserve. These patients resemble individuals with T2D obesity phenotype and account for 50% of all KPDM (5).

Nalini et al. further divided the A–β+ KPDM group into unprovoked vs. provoked DKA. Approximately 50% of the A–β+ subgroup present with new onset diabetes and DKA without clinically evident precipitating factors. The rest of this subgroup has a history of long-standing diabetes and develops ketoacidosis in the setting of an acute illness or non-compliance with antidiabetic treatment. In a cohort studied by Nalini et al. (8), the majority of unprovoked A–β+ KPDM were male, African American, with greater BMI, while the majority of provoked A–β+ KPDM were female and Hispanic similar to our patient (8). In addition, the unprovoked A–β+ KPDM have greater improvement in β- cell function, better glycemic control and twice the rate of insulin discontinuation 12 months after DKA event. The unprovoked group had association with a type 1 diabetes resistance allele DQB1^*^0602 vs. the provoked group had increased frequency of type 1 susceptibility alleles DQB1^*^0302 and DRB1^*^04. These specific immunological factors may contribute to the decline in beta function seen in the provoked KPDM group. These data suggest patients with unprovoked or provoked A–ß+ KPDM have distinct underlying mechanisms of ß cell dysfunction. Approximately 60% of all A-β+ KPDM patients are insulin dependent after 10 years (9).

There is no gold standard for the diagnosis of KPDM. However, islet antibodies and C-peptide levels should be obtained for all patients with suspected KPDM. C-peptide reflects endogenous insulin secretion by pancreatic β cells; it can be measured fasting or with a stimulation test. Islet-specific pancreatic antibodies include antibodies against glutamic acid decarboxylase (GAD 65 Ab) and antibodies against islet cells (ICA Ab). Assessment of both β- secretory reserve and β-cell autoimmunity should be performed after complete resolution of DKA to minimize the effects of glucose toxicity. Zinc transporter 8 antibody was not checked in our case. It is an additional marker of autoimmune diabetes and when combined with antibodies to insulin, GAD, or protein tyrosine kinase IA-1 it can raise the detection rate of autoimmunity to 94% in new onset cases of T1DM. Zinc transporter 8 antibody does decrease with increasing age, but may have been helpful in this case (10). In the Aβ KPDM classification, β+ is defined as a fasting C-peptide concentration >1 ng/ml and peak serum C-peptide concentration after glucagon stimulation >1.5 ng/ml (measured at 5 and 10 min after intravenous injection of 1 mg glucagon) (7).

Our patient has a random C-peptide of 1.36 ng/mL non-fasting, which does correspond to β cell secretory reserve but not as accurately as a fasting or stimulated C-peptide. The aforementioned cut offs predict β- cell function after six and 12 months and glycemic control after 1 year. A high ratio (>11) of fasting C- peptide (in nmol/L) to glucose (ml/L) predicts the potential safe discontinuation of insulin (7). Patients with long-standing diabetes and provoked DKA have a decreased chance of insulin discontinuation (7). Nalini et al. demonstrated a lesser ß cell improvement and poorer glycemic control in the provoked A-β+ KPDM group than in the unprovoked group. Our patient had a >10-year history of diabetes and presented with provoked DKA making it likely she will be insulin dependent.

DKA has been reported post bariatric surgery in type 1 and type 2 diabetes. DKA can occur post bariatric surgery in T2D but is uncommon and mild (11). Aminian et al. reported 12 patients who developed DKA after bariatric surgery at an academic center from January 2005 to December 2015. Sixty-six percent of patients who developed DKA had T1D and thirty three percent had T2D. The median interval between bariatric surgery and DKA development was 12 days (range was 0–61 days). Three out of the four patients with T2D who developed DKA were on insulin prior to surgery. Two out of the three patients had been on insulin for many years and the discontinuation of insulin post operatively was thought to be the cause of DKA. Additional precipitation factors included infection and poor oral intake (11). Arminian et al., recommend optimizing glycemic control before surgery, administering basal insulin on the morning of surgery, and using perioperative infusion protocols in the perioperative period to prevent post-operative DKA. Our patient did not have optimal glycemic control prior to surgery and also had a history of non-compliance with medications putting her at high- risk of post-operative complications. There are currently no case reports specifying KPDM and DKA post bariatric surgery.

Infection is the most common precipitating event in DKA. Our patient initially had a white blood cell count (WBC) of 26.5 U/KL concerning for infection at presentation of DKA. Repeat WBC within 24 h was 16.9 U/KL with 85% polymorphonuclear leukocytes. Although it is common to have leukocytosis with DKA, a WBC >25.0 U/KL is more likely to be related to occult infection (3). Although there was no definitive infection found and WBC decreased and normalized by the time of discharge, a viral infection was suspected as the precipitating event in our patient in addition to the discontinuation of insulin.

In addition, the patient had a history of using empagliflozin, a sodium glucose cotransporter- 2 (SGLT2) inhibitor. SGLT2s can cause DKA and usually present as euglycemic and moderate hyperglycemic DKA (11). Our patient reported stopping SGLT2 ~3 months prior to presentation, making this precipitant less likely.

Although bariatric surgery can lead to diabetes remission, it is essential to know the type of diabetes a patient has prior to bariatric surgery. KPDM is more common in the non-Caucasian population and in particular provoked DKA is more prevalent in Hispanics. This case demonstrates caution must be used in discontinuation of insulin post bariatric surgery for non-Caucasian patients on insulin for many years despite a T2D phenotype. Patients need to be educated on potential for DKA post-operatively and signs and symptoms of DKA specially with discontinuation of insulin post operatively or other precipitants such as infection. Our patient had a classic T2D phenotype but given her presentation of provoked DKA and history of insulin use prior to bariatric surgery it is very likely she will be insulin dependent to avoid DKA even with a significant decrease in insulin requirements.

## Author Contributions

IR critically reviewed for important intellectual content and wrote the first draft. CA contributed to writing the manuscript. IR and CA provided approval for publication of the content, agreed to be accountable for all aspects of the work in ensuring that questions related to the accuracy or integrity of any part of the work are appropriately investigated and resolved.

### Conflict of Interest Statement

CA has participated on advisory boards for Nutrisystem, Zafgen, Sanofi-Aventis, Orexigen, EnteroMedics, GI Dynamics, Scientific Intake, Gelesis, Novo Nordisk, SetPoint Health, Xeno Biosciences, Rhythm Pharmaceuticals, Eisai, Scientific Intake and Takeda. CA has received research funding from Aspire Bariatrics, GI Dynamics, Orexigen, Takeda, the Vela Foundation, Gelesis, Energesis and Coherence Lab. CA has participated on the Takeda Speakers Bureau for the medication Contrave. CA owned stock in Science-Smart LLC. The remaining author declares that the research was conducted in the absence of any commercial or financial relationships that could be construed as a potential conflict of interest.

## Abbreviations

DKA: diabetic ketoacidosis
RYGB: roux-en-y gastric bypass
SGLT2: SGLT 2 inhibitor
T2D: type 2 diabetes
T1D: type 1 diabetes
BMI: body mass index
KPDM: ketosis-prone diabetes mellitus
WBC: white blood cell count.
